# A Proof of Concept Study of Using Machine-Learning in Artificial Aortic Valve Design: From Leaflet Design to Stress Analysis

**DOI:** 10.3390/bioengineering6040104

**Published:** 2019-11-08

**Authors:** Liang Liang, Bill Sun

**Affiliations:** 1 Department of Computer Science, University of Miami, Coral Gables, FL 33146, USA; 2 Walton High School, Marietta, GA 30062, USA; billsun9@gmail.com

**Keywords:** artificial heart valve, transcatheter aortic valve, finite element analysis, machine learning, deep neural network

## Abstract

Artificial heart valves, used to replace diseased human heart valves, are life-saving medical devices. Currently, at the device development stage, new artificial valves are primarily assessed through time-consuming and expensive benchtop tests or animal implantation studies. Computational stress analysis using the finite element (FE) method presents an attractive alternative to physical testing. However, FE computational analysis requires a complex process of numeric modeling and simulation, as well as in-depth engineering expertise. In this proof of concept study, our objective was to develop machine learning (ML) techniques that can estimate the stress and deformation of a transcatheter aortic valve (TAV) from a given set of TAV leaflet design parameters. Two deep neural networks were developed and compared: the autoencoder-based ML-models and the direct ML-models. The ML-models were evaluated through Monte Carlo cross validation. From the results, both proposed deep neural networks could accurately estimate the deformed geometry of the TAV leaflets and the associated stress distributions within a second, with the direct ML-models (ML-model-d) having slightly larger errors. In conclusion, although this is a proof-of-concept study, the proposed ML approaches have demonstrated great potential to serve as a fast and reliable tool for future TAV design.

## 1. Introduction

In the past 15 years, transcatheter valve devices have experienced explosive growth and development in the field of transcatheter cardiovascular therapy. Transcatheter aortic valve replacement (TAVR), first implanted in a human in 2002 by Alan Cribier [1], has been performed for over 350,000 patients in over 70 countries [2]. TAVR is now the standard-of-care treatment for inoperable and high-risk patients with severe aortic stenosis [3], and has recently been expanded to intermediate risk patients [4].

A myriad of new transcatheter valve devices with innovative designs have been developed and undergone pre-clinical and clinical trials [5,6]. Some devices (e.g., Edwards SAPIEN TAVR series), have had more success than others [7,8], but many of the new devices were met with dismal results and did not reach the CE mark or US FDA approval [9]. Artificial heart valves are FDA Class III medical devices that require significant investment and years of development from their concept designs to commercialization. In such a time-consuming and expensive process, it is paramount that any faulty valve design can be detected and corrected as soon as possible before proceeding to pre-clinical and clinical trials.

TAVR devices are typically comprised of either glutaraldehyde-treated bovine pericardium or porcine pericardium valve leaflets [10], similar to surgical pericardial valves (e.g., Carpentier-Edwards Perimount valve), which are known to have limited durability due to calcification and/or mechanical fatigue-induced structural valve deterioration of the leaflets [11,12]. Currently, at the device development stage, TAVR durability is primarily assessed through time-consuming accelerated wear tests (AWT) or expensive animal implantation studies. Based on ISO standards (e.g., ISO 5840-3) [13], the FDA mandates 200 million cycles of AWT tests before any commercial TAVR is approved. Though these bench tests [14,15,16,17] and animal studies [18] provide critical insight regarding valve function and integrity over time, when many design iterations are necessary to find an optimal design, such approaches could be cumbersome or even prohibitive, because many physical valves have to be fabricated and tested.

Stress computation using finite element analysis (FEA) offers an attractive alternative that has been increasingly utilized to evaluate native [19,20] and bioprosthetic valve mechanics [21]. Using patient-specific geometries, Grande-Allen et al. [22,23,24] developed some early FE models to study aortic valve mechanics under valvular insufficiency and Marfan syndrome conditions, although the leaflets were modeled using linear elastic properties. Nonlinear anisotropic material properties of native valve [25,26,27] and pericardial leaflets [28,29] have been experimentally characterized using biaxial testing techniques and incorporated in FE simulations [30,31,32]. For TAV FE analysis, Li and Sun [31] and Smuts et al. [33] studied the impact of TAV leaflet materials, made from bovine, porcine and kangaroo pericardium, on the leaflet stress distribution. Sun et al. [30] investigated the impact of elliptical TAV deployment due to severe aortic calcification on valve function. Auricchio et al. [34], Gunning et al. [35], and Morganti et al. [36] have also investigated TAV leaflet distortion within a realistic, non-circular aortic root; the impact of incomplete TAV expansion on leaflet stress and strain distributions was investigated by Abbasi et al. [37], and TAV fatigue damage analysis was performed by Martin and Sun [12].

However, computational study requires a lengthy and complex process of numeric modeling and simulation, as well as engineering expertise. A potential paradigm-changing solution to the bottleneck is to incorporate machine learning (ML) algorithms [38,39] to expedite and simplify the computational biomechanical analysis process. We have recently demonstrated that machine learning techniques can be applied in a variety of biomechanical analyses [40,41,42,43,44,45,46], which include the reconstruction of the native aortic valve from three-dimensional (3D) computed tomography images [43] and the estimation of stress distribution from FEA [44]. With these promising results, in this proof of concept study, we investigated, for the first time, the use of ML techniques as a surrogate for geometry and stress analysis of TAV design. Given valve design parameters as the input, our goal was that the ML-models (i.e., deep neural networks (DNNs)), once trained on existing FEA results, could accurately estimate the deformed geometry of the TAV leaflets and the associated stress distributions, within a second. To our knowledge, this is the first study to use ML to facilitate TAV design.

## 2. Materials and Methods

**Figure 1:**
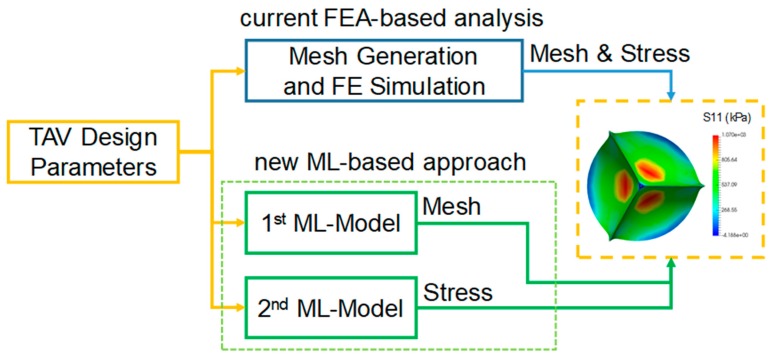
Comparison of the FEA-based approach and the ML-based approach. FEA: finite element analysis. ML: machine learning. TAV: transcatheter aortic valve.

In this study, we developed an ML approach to facilitate TAV design. As illustrated in Figure 1, the “current FEA-based analysis” begins with the three requisite TAV design parameters, generates the FE mesh, and conducts two FE simulations: The 1st FE simulation is for leaflet mounting and the 2nd FE simulation for obtaining the deformed shape and stress distribution of the valve. In the “new ML-based approach,” we will use two ML models, one to model the shape deformation and the other to model the stress distribution to achieve similar results to those from the FEA-based approach.

### 2.1. Data Preparation

A TAV design typically consists of two important components: the leaflet design and the stent design. Although a TAV device consists of three free-form leaflets mounted on a stent, the valve leaflet design begins with a two-dimensional (2D) structure. TAV leaflets are fabricated from a flat sheet of chemically-treated bovine or porcine pericardial tissue. A large, flat pericardial tissue sheet is laser-cut into a specific 2D leaflet shape. A valve assembler carefully stitches three identical flat leaflets onto a stent frame using sutures. As shown in Travaglino et al. [47], for the stent design, the only important geometric information related to the 2D leaflet design is the stent suturing line (*SSL*), by which the 2D leaflet is attached to the circular stent frame to form a 3D leaflet shape. Thus, the specific stent design is not necessary for valve leaflet stress analysis, as long as the *SSL* on the stent is given.

This valve fabrication process can be computationally replicated and simulated. The first step is to create a 2D FE model of the flat leaflet. The sewing process can then be mimicked in a FEA simulation by applying displacement boundary conditions to mount the 2D leaflets on the stent (rigid) based on the *SSL* geometry. As a result, a 3D TAV model is generated, and the valve deformation and stress distribution can be investigated. The processes of obtaining 2D leaflet geometries from leaflet design parameters and performing FE simulations to obtain 3D leaflet deformation and stress distribution have been described in Li and Sun [48] and Travaglino et al. [47]. For completeness, we recap the main equations and the processes below.

#### 2.1.1. Obtain 2D Leaflet Geometries from Design Parameters

**Figure 2:**
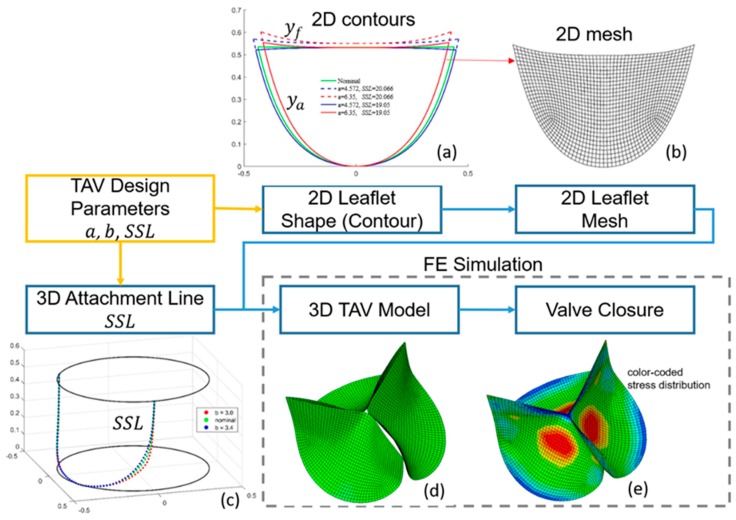
The process of current FEA-based analysis for TAV design. 2D: two-dimensional; 3D: three-dimensional. Examples of 2D designs are shown in (**a**). Each 2D design is converted to a 2D mesh shown in (**b**). The *SSL* designs are shown in (**c**). The assembled 3D leaflets are shown in (**d**). The valve closure simulation result is shown in (**e**).

**Figure 3:**
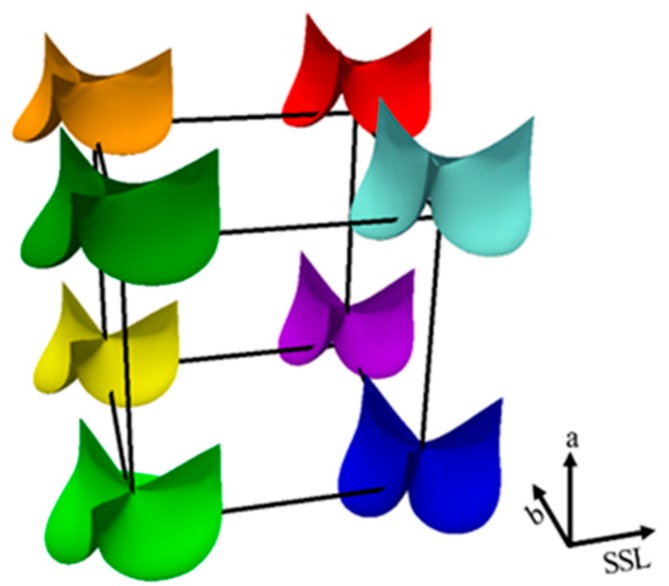
The extreme leaflet shapes visualized in the space of valve design parameters.

Briefly, as shown in Figure 2, a 2D TAV leaflet typically has a scallop shape, with the attachment edge ($y_{a})$ on the bottom and the free edge ($y_{f})$ on the top (Figure 2a). These two curves can be parameterized using two functions [47]:

(1) $$y_{a}=ae^{0.1053x^{2}}$$

(2) $$y_{f}=h\left(1-\frac{e^{\left(x^{3}-\;m^{3}\right)}-1}{e^{-\;m^{3}}-1}\right)\;$$

where m = 10.9 mm. Parameters *a* and *h* are to be determined. With a 2D shape (contour) of a leaflet, a planar leaflet mesh can be created in 2D (Figure 3). The parameter *h* in Equation (2), which determines the height of the free edge in 2D space, is defined as [47]:

(3) $$h=h_{nom}+r\left(SSL-SSL_{nom}\right)$$

where the values of nominal height ($h_{nom})$, nominal *SSL* ($SSL_{nom})$, and *r* are held constant at 13.3 mm, 19.1 mm, and 9.6 mm, respectively. *SSL* is allowed to vary. The shape of the *SSL* on the stent (Figure 2c) in 3D space is defined in cylindrical coordinates by the function [47]:

(4) $$z=pe^{b\theta }$$

where *p* is held constant at 0.20 mm and the value of *b* varies to control the attachment edge shape. For each TAV design, the value of *SSL* length will be conserved between 2D and 3D space.

The three TAV design parameters, {*a*, *b*, *SSL*}, in this study, were confined in feasible ranges: a is within the range of 4.57 to 6.35 mm; b is within the range of 3.0 to 3.4; SSL is within the range of 18.4 to 20.1 mm. A total number of 984 different TAV leaflet geometries were generated and studied within the design space [47].

#### 2.1.2. Obtain Deformed 3D Leaflet Geometries and Stress Distributions from FE Simulations

Given a TAV 2D leaflet design, a FE mesh of 1381 nodes for each of the leaflet was generated. Two FE simulations (Abaqus/Standard 6.16) were performed [47]: (1) the first FE simulation to virtually mount the 2D leaflets on a 3D stent frame; (2) the second FE simulation to apply 120 mmHg pressure to the 3D leaflets to obtained the deformed geometries and stress distribution.

A fiber reinforced hyperelastic material model [49] modified from the GOH model [50] was used to characterize the mechanical response of TAV leaflets. The strain energy function can be expressed as:

(5) $$W=C_{10}\left\{\exp\left[C_{01}\left({\bar{I}}_{1\;}-3\right)\right]-1\right\}+\frac{k_{1}}{2k_{2}}\overset{2}{\underset{i=1}{\sum }}\left\{\exp\left[k_{2}{\left({\bar{I}}_{4i}-1\right)}^{2}\right]-1\right\}+\frac{1}{D}{\left(J-1\right)}^{2}$$

where C_10_, C_01_, k_1_, and k_2_, are material parameters; ${\bar{I}}_{1}$ and ${\bar{I}}_{4i}$ are deviatoric strain invariants; D is a constant to enforce incompressibility. The material parameters in this model were prescribed based on previous planar biaxial mechanical testing experiments on porcine pericardium [10]: $C_{01}=13.48,C_{10}=2.196,\;k_{1}=22.14,\;k_{2}=107.27,\;\kappa =1.16\times 10^{-7},\;\theta =7.81$.

FE simulations of TAV valve closure were performed in Abaqus. The nodes of the attachment edge were kept fixed with zero displacement to mimic the tissue’s connection to a rigid stent. The coefficient of friction between the leaflets was set to be 0.1 [47]. Valve closure was achieved by applying a pressure of 120 mmHg to the aortic side of the leaflets [48,51]. Details of FE simulations and the results can be found in the work of Travaglino et al. [47], and similar works can be found in [48,51]. A total number of 984 different TAV leaflet geometries and associated stress fields were obtained from Travaglino et al. [47] and used in this study.

The extreme leaflet design shapes in the design space of (*a, b, SSL*) are visualized in Figure 3. Each of the three design parameters has a min value and a max value (see 2.2.1), and therefore, there are eight extreme leaflet designs corresponding to the eight combinations of these min/max parameters. It should be noted that the FE analysis of TAV was not performed in this study. The goal of this study is to utilize the existing, published FE results to develop ML models, which will be trained on these FE results, to predict the FE results that are not in the training datasets, as shown in the cross-validation process.

### 2.2. The ML-models

Since geometric and material symmetry can be assumed for a TAV with three leaflets, the deformed geometry and the associated stress field of only one leaflet need to be predicted. The FE mesh of a leaflet has a total of 1381 nodes; thus, to predict the deformed geometry, a numerical vector of 4143 (=1381 × 3) numbers for the three coordinates (i.e., x, y, and z) of the 1381 nodes need to be obtained. Similarly, for the stress field of the leaflet, the in-plane stress components (S11, S12, and S22) of 1381 nodes (4143 numbers again) need to be predicted.

The architecture of the ML models is designed such that the deformed leaflet geometry and stress field will be predicted separately by two ML-models with the same neural network structure (i.e., numbers of neurons and layers). In this study, neural networks are used as nonlinear regressors, which are not based on any mechanical principles, and since stresses and geometries are completely different physical quantities, it is straightforward to develop two separate regressors. The 1st ML-model takes as input a set of TAV design parameters and outputs the deformed geometry. The 2nd ML-model takes as input the same set of TAV design parameters and outputs the stress distribution on the deformed geometry.

#### 2.2.1. The Autoencoder-based ML-models

**Figure 4:**
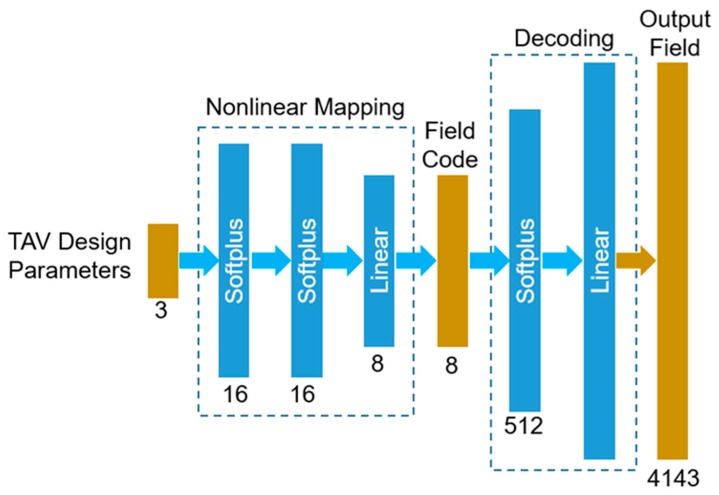
The structure and data flow of the autoencoder-based ML-models.

As shown in Figure 4, the (1st or 2nd) autoencoder-based ML-model, named as ML-model-a, takes the TAV design parameters and converts them to a field code of 8 numbers. The field code is then decoded into the output field of 4143 numbers. A relatively small neural network was designed to map the TAV design parameters {a,b,SSL} to the field code $\left\{\beta_{1},\ldots ,\beta_{8}\right\}$, establishing functions $\beta_{n}=f\left(a,b,SSL\right)$ for every *n = 1,…,8*. This nonlinear mapping module has two hidden layers, each of which has 16 Softplus units, and it has an output layer with eight linear units. A relatively large neural network was designed to decode the field code of eight numbers into the stress field or the deformed geometry. This decoding module has a hidden layer with 512 Softplus units and an output layer with 4143 linear units.

As there are three input parameters and 4143 output variables but only 984 input-output data pairs, there is a potential overfitting issue under supervised learning [52]. Autoencoders were built under unsupervised learning to obtain the decoding modules, and then the nonlinear mapping modules were trained in a supervised manner; finally, the entire ML-models (1st and 2nd) were fine-tuned.

**Figure 5:**
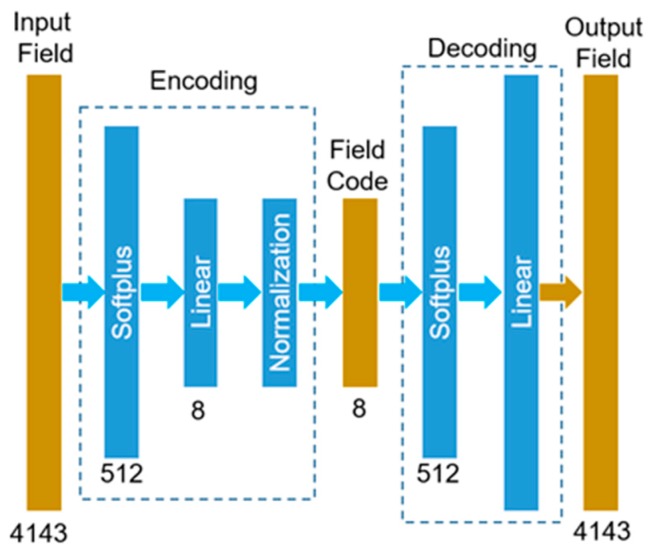
The structure and data flow of an autoencoder.

Each autoencoder [53] is a neural network to obtain a compact representation (the field code) of the input (a field), which is dimensionality reduction with constrained capacity to help to reduce the chance of overfitting in machine learning. An autoencoder takes as input a set of field data (shape or stress), compresses the data into code (a few numbers), and then tries to reconstruct the input field data from the code. As a result, a compact representation (i.e., code) of the input is obtained. The structure and data flow of the autoencoder is shown in Figure 5. Basically, the autoencoder compresses the input field data into field codes (eight numbers) through three layers (Softplus, Linear, and Normalization), and then it tries to recover the field (output) from the field code through two layers (Softplus and Linear). In total, two autoencoders with the same structure were built: one for the 1st ML-model-a to predict deformed geometries and the other for the 2nd ML-model-a to predict the stress fields. After the two autoencoders were developed on training data, the decoding modules of the autoencoders were copied into the 1st and 2nd ML-models-a.

#### 2.2.2. The Direct ML-models

**Figure 6:**
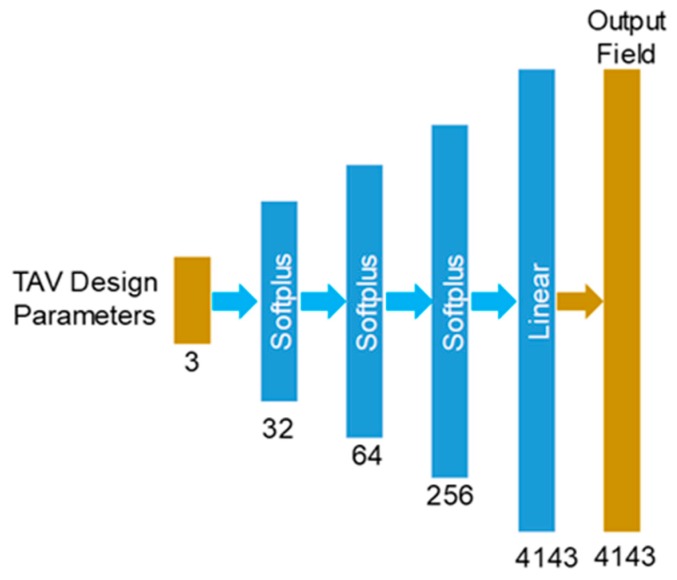
The structure and data flow of each direct ML-model.

As a comparison to the autoencoder-based ML-models, we used two feedforward DNNs for geometry and stress predictions. The two DNNs are named 1st ML-model-d, and 2nd ML-model-d, respectively. Figure 6 shows the structure of each ML-model-d: it has three hidden layers (32, 64, and 256 Softplus units) and one output layer (4143 linear units).

#### 2.2.3. Implementation of the ML-models

The ML-models were implemented using Keras [54] with Tensorflow backend [55]; and the Adamax optimization algorithm [56] was used to find the optimal parameters of the neural networks. We used the default values of the parameters in Adamax optimizer in Keras [54]. Mean Squared Error (MSE) loss was used to measure the discrepancy between the predicted stresses/shapes and the stresses/shapes from FEA. A computer server with Nvidia GTX 1080 Ti GPU was used for model training. After training, the processing speed of each model was measured by using an Intel i7 3.6GHz quad-core CPU only.

#### 2.2.4. Evaluation of the ML-models

The performance of the ML-models was evaluated using Monte Carlo cross validation to test whether the trained models can accurately output the field data. In each round of the cross validation, ρ% of the valve designs and the corresponding shape and stress data were randomly selected as the training data to determine the internal parameters of the ML-models; and the trained ML-models were applied to the remaining (100–ρ)% of the data (i.e., the testing data that was not seen by the ML-models during training) to obtain performance measures. ρ was varied from 50 to 90. For a fixed ρ, the training-testing process was repeated 100 times to obtain the mean and standard deviation of each performance measure. It takes about one hour to complete one round of cross-validation and associated calculations.

(a) Evaluation of the 1st ML-model to predict the deformed shapes of TAV leaflets:

Four performance metrics were used to evaluate the accuracy of the shapes output from the ML-model: mean error (MeanE), normalized mean error (NMeanE), maximum error (MaxE), and normalized maximum error (NMaxE). MeanE is defined by:

(6) $$MeanE=\frac{1}{N}\sum_{i=1}^{N}||X_{i}-{\tilde{X}}_{i}||$$

where $X_{i}$ is the 3D position vector of node-*i* of the “true” TAV mesh from FEA, and ${\tilde{X}}_{i}$ is the corresponding position vector estimated by the ML-model. ||.|| is the Euclidean norm. *N* is the number of nodes of a TAV leaflet mesh. NMeanE is defined by:

(7) $$NMeanE=\frac{MeanE}{R}\times 100\%$$

where R is the radius of the circumcircle determined by the three commissures on the “true” TAV. MaxE is defined by:

(8) $$MaxE=max\left\{||X_{i}-{\tilde{X}}_{i}||,i=1,2,3,\ldots ,N\right\}$$

NMaxE is defined by:

(9) $$NMaxE=\frac{MaxE}{R}\times 100\%$$

(b) Evaluation of the 2nd ML-model to predict the stress fields on TAV leaflets:

Four performance metrics were used to evaluate the accuracy of the stress fields output from the ML-model: mean error (MeanE) and normalized mean error (NMeanE), peak stress error (MaxE), and normalized peak stress error (NMaxE). MeanE is defined by:

(10) $$MeanE=\frac{1}{N}\sum_{i=1}^{N}\left|S\left(i\right)-\tilde{S}\left(i\right)\right|$$

where S(i) is a stress value at node-*i* computed from FEA, and $\tilde{S}\left(i\right)$ is the corresponding stress estimated by the ML-model, and |.| denotes the absolute value. NMeanE is defined by:

(11) $$NMeanE=\frac{MeanE}{\left|\max\left\{S\right\}-\min\left\{S\right\}\right|}\times 100\%$$

where max{S} and min{S} are the maximum and minimum stress values from FEA, respectively.

To compare peak stresses, MaxE is defined by:

(12) $$MaxE=\left|\max\left\{\left|S\right|\right\}-\max\left\{\left|\tilde{S}\right|\right\}\right|$$

where max{|S|} is the peak stress output from FEA, and $\max\left\{\left|\tilde{S}\right|\right\}$ is the corresponding peak stress estimated by the ML-model. NMaxE is defined by:

(13) $$NMaxE=\frac{MaxE}{\max\left\{\left|S\right|\right\}}\times 100\%$$

By substituting $S_{11}$ or $S_{22}$ or $S_{12}$ for S in Equations (10–13), we can obtain error metrics for different stress components.

## 3. Results

The autoencoder-based ML-models and the direct ML-models were trained and tested on a dataset of 984 TAV outputs from FE simulations. The data from FE simulations was considered the ‘ground-truth’, which was compared to the corresponding output from the ML-models. The effect of ρ on the accuracy of predicted TAV leaflet stress fields is shown in Table 1. The performance measures for TAV stress field and deformed shape predictions are reported in Table 2 (ρ = 90) and Table 3 (ρ = 90), respectively.

It can be seen from Table 2 that the percentage errors from both ML-models are very small, indicating that both ML-models can make accurate predictions. The direct ML-models (ML-model-d) have slightly larger errors. Thus, the direct ML-model was not used for shape prediction. On average, the magnitude of S11 is larger than S12 and S22. Since in the loss function, the three stress components have the same weight, and therefore, the prediction error of S11 is higher.

**Figure 7:**
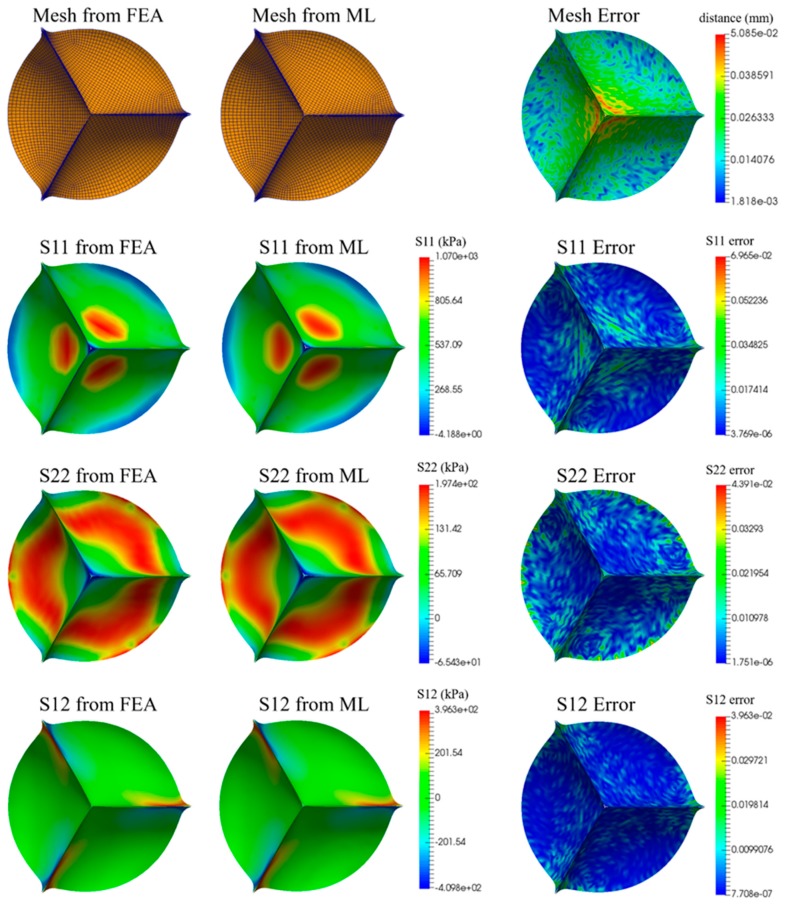
Comparison of deformed shape (i.e., mesh, distance errors are color-coded) and stress distributions (three components: S11, S22, S12) from FEA and ML for one representative example. The relative stress error at node-*n* is defined as $\tilde{S}\left(n\right)/\left|\max\left\{S\right\}-\min\left\{S\right\}\right|$, where $\tilde{S}\left(n\right)$ is from the ML model.

The result of a representative TAV design is shown in Figure 7. The field data were visualized using the open source software Paraview [57]. The overall distributions of stresses from the ML-models are very similar to those from FE simulations, although there are some subtle differences. The errors from deformed shape prediction are shown in color map, which are very small. The performance measures are reported in Table 4.

Given an input set of TAV design parameters, the trained ML-models can output the required fields within one second (excluding initialization and file IO time) on a PC with an Intel i7 3.6GHz quad-core CPU and 32GB RAM. As a comparison, it could take a day to generate the TAV mesh from a set of TAV design parameters and to perform FE simulation on an eight-core computer cluster. If numerical convergence issues are encountered, the process could take much longer and may have to be resolved by human experts through trial and error.

**Figure 8:**
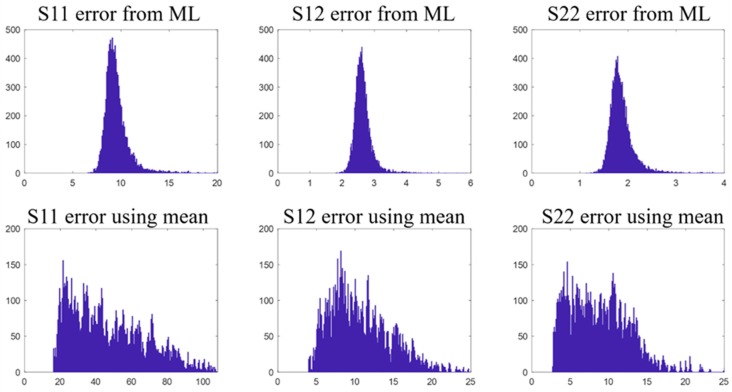
The top row shows the MeanE (kPa) distributions/histograms of S11, S12, and S22 predicted by the 1st ML-model-a. The bottom row shows the MeanE (kPa) distributions of S11, S12, and S22 predicted by using the mean distributions.

**Figure 9:**
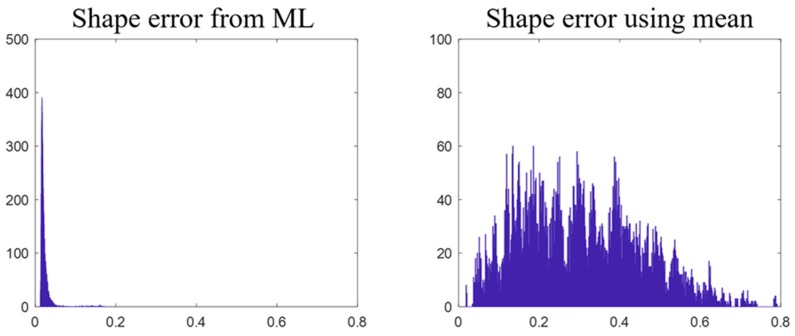
The left plot shows the MeanE (mm) distribution /histogram of shapes predicted by the 1st ML-model-a. The right plot shows the MeanE (mm) distribution of shapes predicted by using the mean shape.

We also studied whether the mean stress distributions of S11, S12, and S22 can predict the stress distributions of different valve designs, and whether the mean deformed-shape can predict the deformed-shapes of different valve designs. Cross validation (Section 2.2.4) was used for the evaluation. In each round of the cross validation, 90% of the valve designs and the corresponding shape and stress data were randomly selected as the training data to compute the mean stress distributions and the mean deformed-shape as if they were “predictive models” that always output the mean stress-distributions and the mean deformed-shape for any inputs; and the “predictive models” were applied to the remaining 10% of the data to calculate MeanE. The process was repeated 100 times. The results are shown in Figure 8 and Figure 9, which clearly reveal the large variation among the data.

## 4. Discussion

In recent years, artificial intelligence has been increasingly utilized in various applications that impact the society. Application of AI tools in engineering design can be dated back to the 1980s when knowledge-based expert systems were first used to automate engineering design processes [58]. In the 1990s, fuzzy logics and neural network were applied to engineering control and design in various systems, such as the computer integrated manufacturing systems [59]. Recently, Deep Learning [38,39,60], a machine learning technique utilizing deep neural networks, has garnered enormous attention in the fields of machine learning and artificial intelligence. As universal function approximators [52,61,62,63,64], DNNs are capable of modeling complex, nonlinear relationships between input and output variables. Thus, given adequate data for training such as the design parameters and stress field data, DNNs could offer an end-to-end solution to directly link the design parameters to the required output, bypassing the complex computational FE model setup and simulation. As demonstrated by us [44] and others [65,66,67,68,69], different DNNs have been developed to speed-up or substitute FEA and computational fluid dynamics (CFD) simulations.

Since the TAV design data (stress fields and deformed geometries) was generated through a series of rigorous engineering analyses using mathematic equations and FE analyses, the paired input-output training data sets do not contain large noises, which reduced the possibility of overfitting. As a result, the autoencoder-based ML-models and the direct ML-models could achieve similar performance (Table 2). As shown in Table 1, when ρ decreased, the prediction errors of the direct model increased slightly whereas the autoencoder-based ML-models were less affected. The developed ML-models were evaluated through cross validation, where an average discrepancy of 0.21% was achieved between FE-computed and ML-predicted geometries, and average discrepancies of 0.98%, 0.71%, and 0.36% were achieved between FE-computed and ML-predicted distributions of stress components: S_11_, S_22_, S_12_, respectively. These results suggest that DNNs can effectively serve as a surrogate of FEA because the noise in the training data from FEA had negligible effect on the output.

In this study, we selected the TAV design space confined by three parameters, *a, b* and *SSL*. The extreme TAV shapes at the boundaries of the design space are depicted in Figure 3. By increasing the computing power and amount of TAV design data, we can enlarge the design space to explore different stent and leaflet shapes that are not included in the current study. Within a feasible design space, the predictability or the “intelligence” of the ML-models for a new design (not previously calculated from FEA) makes ML-models more efficient than the performing FEA for individual new designs. We hope that the success of this proof-of-concept study could enable a more widespread use of ML-techniques to expedite the artificial valve design process.

In this study, the following limitations should be taken into consideration when interpreting our results: (1) FEA was not performed in this study, and FEA results were taken from an existing, published data [47]. Thus, all the limitations associated with the FEA results [47] were inherited into this study, which include but are not limited to: (a) only a set of material properties was used in FE simulations, (b) only static diastolic pressure loading condition was applied to deform the valve geometry. No dynamic systolic deformation (e.g., fluid structure interaction (FSI)) simulations were studied, (c) FEA results may contain numerical errors, and (d) elliptical TAV deployment was not simulated. However, since the purpose of this study was to develop the ML models and test their feasibility in a proof of concept study, these limitations or errors associated with FEA results would not change the conclusions of the study. (2) We note that other types of ML-models (e.g., SVM [70]) could also be used for the tasks in this study. We chose DNNs as the ML-models because DNNs are highly scalable: DNNs can be configured with more layers and units to handle an increasingly large amount of data. For example, in the computer vision field, DNNs have been trained on millions of images for image recognition tasks [71,72]. (3) it is possible to combine the 1st and 2nd ML-models for joint prediction of the stress and shape, which would need more GPU memory. (4) the optimal DNN structure could be obtained by performing neural architecture search [73,74]. Thus, after optimizing structure and training on thousands or even millions of FE simulations in a large design space, the ML-models could be used as a practical tool for TAV design. In order to extend this approach for the stress analysis of diseased native aortic or mitral valve with non-symmetrical geometry, the ML-models need to be re-trained on FEA data of these native valves; otherwise, the prediction will have high errors, which is the nature of machine learning.

## 5. Conclusions

We developed a novel machine learning approach for fast and automatic TAV design and stress analysis. For a set of TAV design parameters, the trained ML-models can output the geometry and stress distributions in one second, consistent with and orders of magnitude faster than the FE-based approach. Although being a proof of concept study, the ML-models have demonstrated a great potential to serve as a fast and reliable tool for TAV design.

## Figures and Tables

**Table 1:** Effect of ρ on the accuracy of predicted TAV leaflet stress fields (ρ = 50, 70, 90).

	ρ = 50	ρ = 70	ρ = 9 0
S_11_ MeanE (kPa) (1st ML-model-a)	8.9218 ± 0.7605	8.8439 ± 0.7364	8.8005 ± 0.7412
S_11_ MeanE (kPa) (1st ML-model-d)	9.6913 ± 1.2928	9.6809 ± 1.2893	9.1142 ± 0.8929

Note: other performance measures follow the same trend.

**Table 2:** Performance measures for the prediction of TAV leaflet stress fields (ρ = 90).

Stress Component	MeanE (kPa)	NMeanE (%)	MaxE (kPa)	NMaxE (%)
S_11_ (1st ML-model-a)	8.8005 ± 0.7412	0.9771 ± 0.1115	15.7456 ± 10.7627	1.5913 ± 1.0787
S_22_ (1st ML-model-a)	1.7251 ± 0.2634	0.7068 ± 0.1026	4.1948 ± 3.7976	1.9873 ± 1.7882
S_12_ (1st ML-model-a)	2.5000 ± 0.1944	0.3638 ± 0.0350	4.3933 ± 3.4736	1.2560 ± 0.9766
S_11_ (1st ML-model-d)	9.1142 ± 0.8929	1.0117 ± 0.1229	16.3978 ± 11.2060	1.6570 ± 1.1235
S_22_ (1st ML-model-d)	1.7809 ± 0.2765	0.7295 ± 0.1071	4.1396 ± 3.7576	1.9622 ± 1.7716
S_12_ (1st ML-model-d)	2.5669 ± 0.2020	0.3734 ± 0.0358	5.3510 ± 4.6305	1.5411 ± 1.3590

Note: the data format in each entry of the table is mean value ± standard deviation.

**Table 3:** Performance measures for the prediction of TAV deformed shapes (ρ = 90).

	MeanE (mm)	NMeanE	MaxE (mm)	NMaxE
Mean Value	0.02164	0.2053%	0.05451	0.5171%
Standard Deviation	0.01235	0.1174%	0.02203	0.2091%

**Table 4:** Performance measures of the ML model tested on a TAV design shown in Figure 7.

	MeanE	NMeanE	MaxE	NMaxE
Mesh	0.02046 (mm)	0.1951%	0.05085 (mm)	0.4850%
Stress S_11_	9.0002 (kPa)	0.8375%	43.9637 (kPa)	4.1070%
Stress S_22_	1.6559 (kPa)	0.6300%	2.8710 (kPa)	1.4545%
Stress S_12_	2.6341 (kPa)	0.3267%	0.9901 (kPa)	0.2416%
